# Tracking under-five mortality from 1990 to 2023: Global, regional, and national trends, inequities, and projections toward achieving SDG Target 3.2 by 2030

**DOI:** 10.1371/journal.pone.0343745

**Published:** 2026-04-01

**Authors:** Guiying Cao, Jue Liu, Min Liu

**Affiliations:** 1 School of Public Health, Peking University, Beijing, China; 2 Key Laboratory of Epidemiology of Major Diseases (Peking University), Ministry of Education, Beijing, China; University of Gondar, ETHIOPIA

## Abstract

**Background:**

In 2015, the UN General Assembly set Sustainable Development Goals (SDGs), in which SDG 3.2 called for all countries to reach an under-5 mortality rate (U5MR) of at least as low as 25 deaths per 1000 livebirths by 2030. As the SDG era enters its final years, it is time to take stock of worldwide trends in U5MRs and progress towards SDG target 3.2. This study aimed to evaluate the time trends in under-5 mortality from 1990 to 2023 with projections towards SDG target 3.2 at the global, regional, and national levels.

**Methods:**

Annual under-5 deaths and U5MR between 1990 and 2023 at the global, regional, and national levels were collected from a dataset complied by the UN Inter-agency group for Child Mortality Estimation. The percentage changes in under-5 deaths and the estimated annual percentage changes (EAPCs) in U5MRS at the global, regional, and national levels were calculated. Trend extrapolation of the EAPCs was used to estimate the year in which locations with a U5MR > 25 deaths per 1000 live births in 2023 would achieve the SDG 3.2 U5MR target.

**Results:**

Globally, the number of under-5 deaths decreased by 63.20%, from 12.98 million in 1990 to 4.78 million in 2023, and the U5MR decreased by an average of 3.18% (95% CI: 3.05%, 3.30%) per year from 93.58 deaths per 1000 livebirths in 1990 to 36.72 deaths per 1000 livebirths in 2023. The global U5MR is expected to achieve SDG target 3.2 by 2035. At the regional level, the U5MR decreased significantly in all SDG regions between 1990 and 2023 but remained above SDG target 3.2 in Sub-Saharan Africa (68.82 deaths per 1000 live births) and Central and Southern Asia (33.23 deaths per 1000 live births) in 2023. Sub-Saharan Africa and Central and Southern Asia are projected to reach the SDG 3.2 target by 2055 and 2030, respectively. At the national level, the U5MR decreased significantly in 192 countries and territories between 1990 and 2023 but increased significantly in Dominica (EAPC = 2.38; 95% CI: 2.18, 2.59) and remained stable in 7 countries and territories during this period. Among the 200 countries and territories worldwide, 133 have already met SDG target 3.2, and 9 are expected to do so by 2030. Of the remaining 58 countries that would not achieve SDG target 3.2 by 2030, 22 would meet it between 2031 and 2040, 10 would meet it between 2041 and 2050, and 25 would meet it after 2050, if the average rate of decline from 1990 to 2023 continues; however, the U5MR would deteriorate in Dominica.

**Conclusions:**

Despite substantial progress in reducing the U5MR worldwide, more than a quarter of countries and territories would not meet SDG target 3.2 by 2030, and nearly half of those countries and territories would meet SDG target 3.2 after 2050. Urgent actions are needed in most regions and countries with high U5MRs, particularly those in sub-Saharan Africa.

## Introduction

The under-5 mortality rate (U5MR) is an important indicator reflecting multiple aspects of societal wellbeing such as access to nutrition and food; basic infrastructure such as housing, water, and sanitation; education; agency; financial security; access to preventive and treatment health services; and future human capital [1]. The survival of children under 5 years of age has been the principal focus of the global child health agenda in recent decades. In 2000, Millennium Development Goal 4 (MDG4) was established to catalyze extraordinary political, financial, and social commitments to reduce U5MR by two-thirds in the period between 1990 and 2015 [2]. The MDGs sparked those who were involved in the 2003 Lancet Child Survival Series to propose the Countdown initiative and pledged to hold regular conferences to “ensuring that there is an overall mechanism for improving accountability, re-energizing commitment, and recognizing accomplishments in child survival” [3]. Countdown to 2015 was launched in 2005, ended in October 2015, and hailed for its substantial contributions to monitoring the progress toward achieving the MDGs. Since 2016, the MDGs have been replaced by the more all-encompassing, albeit less health-focused, Sustainable Development Goals (SDGs) [4]. SDG 3.2 specifically calls to, “By 2030, end preventable deaths of newborns and children under 5 years of age, with all countries aiming to reduce neonatal mortality to at least as low as 12 per 1000 live births and under-5 mortality to at least as low as 25 per 1000 live births.” Countdown to 2015 was relaunched as Countdown to 2030 in 2016 following the publication of the SDGs and the Global Strategy for Women’s, Children’s and Adolescents’ Health 2016–2030. The main aims of Countdown to 2030 are to improve coverage measurement and monitoring, and strengthen the regional and country capacity for evidence generation and use [5]. Countdown to 2015 occupied a unique and critically important niche during the MDG era, and Countdown to 2030 continues to do so in the SDG era, providing the independent monitoring and rigorous analysis that are crucial to accountability.

Over the past several decades, dramatic progress has been made in improving health and reducing the mortality rate of children under 5 years of age globally. The global U5MR has been reduced by nearly half from 93 deaths per 1000 livebirths in 1990–37 deaths per 1000 livebirths in 2022 [6]. However, despite such an extraordinary reduction, the global U5MR is still well above the target of 25 deaths per 1000 livebirths as defined by SDG 3.2. Among the 4.89 million deaths that still occurred among children under 5 years of age in 2022, many were concentrated in vulnerable populations, especially in countries in Sub-Saharan Africa and Southern Asia [6]. The UN Department of Economic and Social Affairs reported that COVID-19 threatens to reverse the progress of SDG 3, including child mortality, as it continues to erode health systems, disrupt routine health services and constrain access to nutritious diets and essential nutrition services [7]. In addition, it is concerning that there are also substantive threats and inequities that jeopardize child health and survival worldwide [5,6,8,9]. Economic trends are of great concern, including slowing economic growth, stalled poverty reduction, and a major debt crisis. In 2021, 25 of 43 countries (58%) with data in sub-Saharan Africa spent more on public external debt servicing than on health [10]. Many countries are affected by armed conflicts, which have resulted in an estimated 507 million children living in or near conflict zones globally in 2022 [10]. Food insecurity has risen during the SDG period, fueled by the COVID-19 pandemic, economic volatility, and armed conflict [11–13]. Climate change, along with its associated consequences of extreme weather events, infrastructure destruction, food insecurity, emerging and re-emerging diseases, and altered disease transmission patterns, poses a severe threat to children’s health [8, 14,15]. These findings remind the world should keep track progress of the reduction of child mortality to reduce inequities and end preventable deaths among children worldwide.

As the SDG era has entered its final years, especially after the COVID-19 pandemic, the global public health and development communities must take stock of progress over the past decades. There has not yet been a comprehensive assessment of the U5MRs in the SDG era, both before and after the COVID-19 pandemic. The selected publications either assessed progress toward the 2030 targets or provided projections to 2030 prior to the COVID-19 pandemic, and none projected the specific years by which countries unlikely to meet the targets by 2030 would achieve them [1,16–20]. Recognizing the urgency of reaching the SDG 3.2 targets by 2030, we used detailed data on under-5 mortality compiled by the UN Inter-agency group for Child Mortality Estimation (UN IGME) to determine the global, regional, and national trends in URMRs between 1990 and 2023, a timeframe covering both the MDG and SDG periods. We further evaluated progress toward SDG Target 3.2 and specifically forecasted when locations with a U5MR over 25 per 1000 live births in 2023 will meet this target under current trends, filling an analytical gap left by routinely published UN reports that lack such year-specific attainment predictions, and providing a more comprehensive evidence base to form the formulation of global and regional targeted interventions and health policies.

## Methods

### Data source

This study used data on annual under-5 deaths and U5MR from 1990 to 2023 by sex and location compiled by the UN IGME. U5MR is the probability of dying between birth and exact age 5, expressed per 1000 live births. The UN IGME formed in 2004 is led by the United Nations Children’s Fund and includes the World Health Organization, the World Bank Group and the United Nations Population Division of the Department of Economic and Social Affairs as full members. UN IGME produces U5MR estimates based on nationally representative data, and newly available data include recently released vital statistics from civil registration systems, results from recent household surveys and censuses, and, occasionally, results from older censuses or surveys not previously available [21]. Civil registration systems collect administrative records of births and deaths prospectively and continuously, which makes this the preferred source of data. For countries without (or with limited data from) well-functioning vital registration systems, complete or summary birth histories of women, collected in surveys and censuses, are often the main source of information on U5MR. This type of data is collected from censuses, Demographic and Health Surveys, and Multiple Indicator Cluster Surveys. Although data availability and advances in analytical methods have been substantially improved over the last three decades, the status quo is by no means satisfactory. For developed countries, estimates of U5MR are typically derived from civil registration systems. For almost all countries in sub-Saharan Africa, estimates of U5MR are derived from household sample surveys and are therefore affected by known biases and sampling errors. In addition, for the great majority of developing countries without well-functioning vital registration systems, estimating levels and trends in U5MR is challenging, not only because of limited data availability but also because of issues with data quality.

To account for the issues related to data quality and the availability of more recent data, UN IGME developed a Bayesian B-splines bias-adjusted model, referred to as the B3 model [22]. In the short term, the B3 model allows for inclusion of information from incomplete vital registration systems. The inclusion of data from alternative data sources and the implementation of novel data collection methods can provide accurate and timely child mortality data and further support child mortality estimation. In the B3 model, the logarithm of U5MR is estimated with a flexible spline regression model. The spline regression model is fitted to all U5MR observations in the country. An observed value for U5MR is considered to be the true value for this parameter multiplied by an error multiplier (i.e., observed U5MR = true U5MR × error multiplier or on the logarithmic scale log[observed U5MR]=log[true U5MR] + log[error multiplier]). The error multiplier refers to the relative difference between an observation and the true value, with the error multiplier equal to 1 (or log[error multiplier] equal to zero) if there is no error. Given the inherent uncertainty in U5MR, UN IGME reported 90% uncertainty intervals (UIs)—i.e., there is a 90% chance that the interval contains the true value. The number of estimated under-5 deaths was calculated using a birth-week cohort method, which is based on the estimated mortality rates, as well as estimates of population numbers by the UN Population Division [19,20]. Under-5 deaths in each calendar year are calculated by summing all the deaths under 5 years of age across all age group cohorts in that year. The 90% UIs for the under-5 deaths were based on the uncertainty in the U5MR estimates and did not account for other sources of uncertainty in its inputs, such as the annual number of livebirths. Specific methods of the UN IGME estimation process for under-5 mortality have been described elsewhere [19,20]. As of January 2024, the database for under-5 mortality contains over 20 400 country-year data points from more than 2300 series across 200 countries and territories that were categorized into 7 SDG regions from 1990 to 2023. Increased empirical data have substantially changed UN IGME estimates for some countries from previous editions, this is partly because the fitted trend line is based on the entire time series of data available for each country. In addition, the UN IGME assesses data quality and excludes data sources with substantial non-sampling errors or omissions from serving as the underlying empirical data in its statistical models. Empirical databases and final estimates are updated annually and are available from the UN IGME web portal, Child Mortality Estimation Info, available at <https://childmortality.org > .

### Statistical analysis

First, we presented the numbers of under-5 deaths and U5MRs with 90% uncertainty intervals (UIs) in 1990 and 2023 at the global, regional, and national levels. Second, we calculated the percentage changes in under-5 deaths and the estimated annual percentage changes (EAPCs) in U5MRs at the global, regional, and national levels to quantify their temporal trends. The percentage changes in under-5 deaths from 1990 to 2023 were calculated via the following equation: percentage change = $\;\frac{\text{Deaths in 2023 }-\text{ Deaths in 1990}}{\text{Deaths in 1990}}\;\times \text{100}\%$. The EAPC is a summary and widely used measure of the rate tend over a specified time interval [23]. A regression line was fitted to the natural logarithm of the U5MR, i.e., y = α + βx + ε, where y = ln (U5MR) and x denotes the calendar year, α is the intercept, β was the regression coefficient on x (calendar year), and ε is the error term. The EAPC was calculated as $\text{100}\times {(e}^{\beta }-\text{1})$ and its 95% confidence interval (CI) can also be obtained from the linear regression model [24]. The trend in rate was reflected in the EAPC value and its 95% CI: rate is in an upward trend when the EAPC and the lower boundary of the 95% CI are positive; conversely, rate is in a downward trend when EAPC and the upper boundary of the 95% CI are negative. We calculated the EAPCs in U5MRs between 1990 and 2023 to reflect their temporal trends in this period. Third, we assumed a log-linear trend in U5MR for trend extrapolation to estimate the year in which locations with a U5MR > 25 deaths per 1000 live births in 2023 would achieve the SDG 3.2 target of U5MR ≤ 25 deaths per 1,000 live births. The year of achievement was estimated based on the EAPC in the U5MR between 1990 and 2023 and the U5MR in 2023. All analyses were conducted with SAS 9.4 (SAS Institute, Inc., Cary, NC, USA). A two-tailed *P* value <0.05 was considered statistically significant. Data visualization was created by ArcGIS Desktop 10.7 (Esri, Redlands, USA) and OriginPro 2022 (OriginLab, Northampton, USA).

## Results

### Global trends in under-5 mortality with projections towards SDG target 3.2

**Fig 1:**
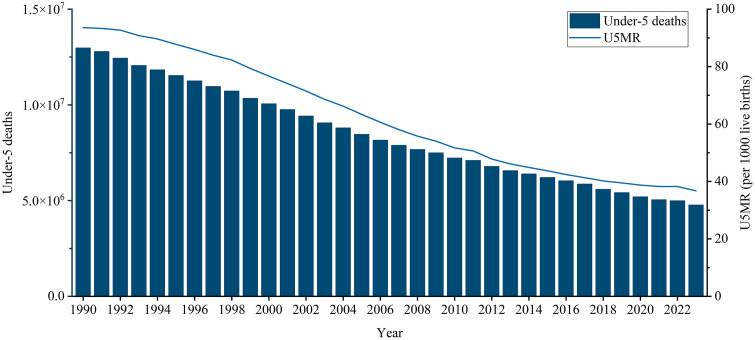
Global under-5 deaths and U5MR, 1990−2023. U5MR: under-5 mortality rate.

Globally, the number of under-5 deaths decreased by 63.20%, from 12.98 (90% UI: 12.80, 13.19) million in 1990 to 4.78 (90% UI: 4.51, 5.33) million in 2023 (Table 1 and Fig 1). The global U5MR decreased by an average of 3.18% (EAPC = −3.18, 95% CI: −3.30, −3.05) per year from 93.58 (90% UI: 92.27, 95.11) deaths per 1000 livebirths in 1990 to 36.72 (90% UI: 34.66, 41.09) deaths per 1000 livebirths in 2023 (Table 1 and Fig 1). The global U5MR is projected to reach the SDG 3.2 target of fewer than 25 deaths per 1000 live births by 2035 (S1 Table). The global number of deaths decreased by 62.06%, from 6.86 (90% UI: 6.76, 6.98) million in 1990 to 2.60 (90% UI: 2.46, 2.90) million in 2023 for male children under 5 years of age and decreased by 64.47% from 6.12 (90% UI: 6.03, 6.22) million in 1990 to 2.17 (90% UI: 2.05, 2.43) million in 2023 for female children under 5 years of age (Table 1). The global mortality rates decreased by 3.06% (EAPC = −3.06, 95% CI: −3.18, −2.94) and 3.31% (EAPC = −3.43, 95% CI: −3.18, −3.22) per year between 1990 and 2023 for male and female children under 5 years of age, respectively (Table 1). For male and female children under 5 years of age worldwide, the mortality rate is projected to reach the SDG 3.2 target by 2038 and 2033, respectively (S1 Table).

**Table 1:** Global under-5 deaths and U5MRs by sex and location in 1990 and 2023 and their trends from 1990 to 2023.

Characteristics	1990		2023		1990-2023	
	Deaths in thousand No. (90% UI)	Mortality rate per 1000 live births No. (90% UI)	Deaths in thousand No. (90% UI)	Mortality rate per 1000 live births No. (90% UI)	Percentage change in deaths (%) No.	EAPC in mortality rate No. (95% CI)
Global	12979.92 (12798.86, 13188.36)	93.58 (92.27, 95.11)	4776.62 (4511.45, 5329.38)	36.72 (34.66, 41.09)	−63.20	−3.18 (−3.30, −3.05)
Sex						
Male	6859.70 (6759.25, 6976.77)	95.82 (94.42, 97.49)	2602.39 (2457.85, 2901.35)	38.95 (36.77, 43.56)	−62.06	−3.06 (−3.18, −2.94)
Female	6120.22 (6028.28, 6224.42)	91.19 (89.83, 92.77)	2174.24 (2049.79, 2428.61)	34.34 (32.37, 38.48)	−64.47	−3.31 (−3.43, −3.18)
SDG region						
Sub-Saharan Africa	3829.48 (3752.63, 3914.30)	181.34 (177.67, 185.49)	2683.63 (2426.77, 3195.37)	68.82 (62.20, 82.20)	−29.92	−3.16 (−3.27, −3.05)
Western Asia and Northern Africa	732.09 (715.55, 750.45)	78.23 (76.44, 80.23)	282.66 (246.77, 339.41)	24.34 (21.23, 29.27)	−61.39	−3.65 (−3.80, −3.50)
Central and Southern Asia	5191.91 (5067.08, 5319.53)	124.85 (121.82, 127.94)	1291.77 (1177.96, 1429.81)	33.23 (30.29, 36.80)	−75.12	−4.09 (−4.19, −3.98)
Eastern and South-Eastern Asia	2361.30 (2250.48, 2493.74)	56.88 (54.19, 60.10)	303.28 (272.52, 349.75)	14.31 (12.83, 16.56)	−87.16	−4.78 (−5.01, −4.55)
Latin America and Caribbean	646.95 (628.92, 666.92)	54.80 (53.27, 56.48)	148.65 (138.79, 164.75)	15.86 (14.81, 17.60)	−77.02	−3.79 (−4.01, −3.56)
Oceania	16.94 (15.83, 18.09)	33.14 (30.95, 35.44)	13.13 (9.43, 18.89)	19.32 (13.87, 27.86)	−22.50	−1.60 (−1.75, −1.44)
North America and Europe	201.26 (198.96, 204.30)	14.26 (14.09, 14.48)	53.50 (52.51, 54.64)	5.07 (4.98, 5.18)	−73.42	−3.20 (−3.30, −3.10)

CI: confidence interval; EAPC: estimated annual percentage change; SDG: Sustainable Development Goals; U5MR: under-5 mortality rate; UI: uncertainty interval.

### Regional trends in under-5 mortality with projections towards SDG target 3.2

**Fig 2:**
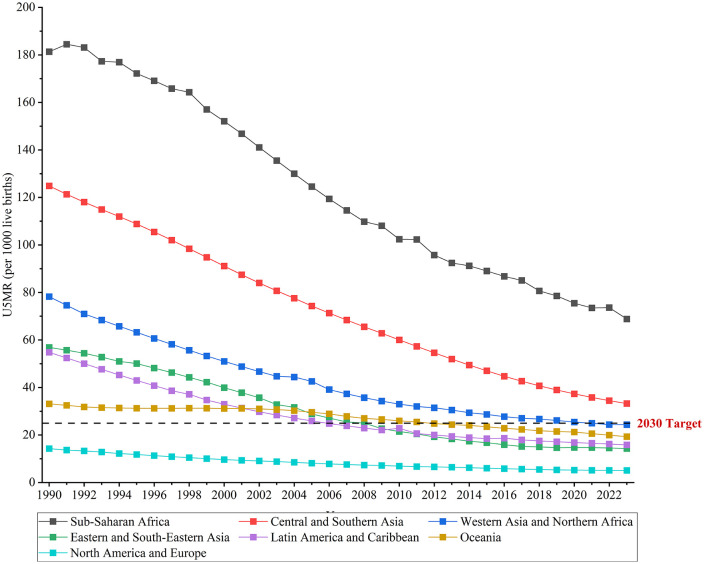
U5MR in SDG regions, 1990−2023. U5MR: under-5 mortality rate; SDG: Sustainable Development Goals.

Across seven SDG regions, most of the global under-5 deaths (83.23%) were concentrated in Sub-Saharan Africa (2.68 (90% UI: 2.43, 3.20) million) and Central and Southern Asia (1.29 (90% UI: 1.18, 1.43) million) in 2023 (Table 1). The number of under-5 deaths decreased in all SDG regions from 1990 to 2023, with the largest decrease in Eastern and South-Eastern Asia (−87.16%), followed by Latin America and Caribbean (−77.02%) (Table 1). All the SDG regions experienced a significant decreasing trend in U5MRs between 1990 and 2023, with the largest decrease in Eastern and South-Eastern Asia (EAPC = −4.78; 95% CI: −5.01, −4.55), followed by Central and Southern Asia (EAPC = −4.09; 95% CI: −4.19, −3.98) and Latin America and the Caribbean (EAPC = −3.79; 95% CI: −4.01, −3.56) (Table 1). In 2023, the U5MR ranged from 5.07 to 68.82 deaths per 1000 live births across SDG regions, with Western Asia and Northern Africa, Eastern and South-Eastern Asia, Latin America and Caribbean, the Oceania, and North America and Europe already achieving the U5MR target in SDG 3.2 (Table 1 and Fig 2). Central and Southern Asia is projected to reach a U5MR of at least 25 deaths per 1000 livebirths by 2030 (S1 Table). The U5MR in Sub-Saharan Africa is projected to reach the SDG 3.2 target by 2055 (S1 Table).

### National trends in under-5 mortality with projections towards SDG target 3.2

**Fig 3:**
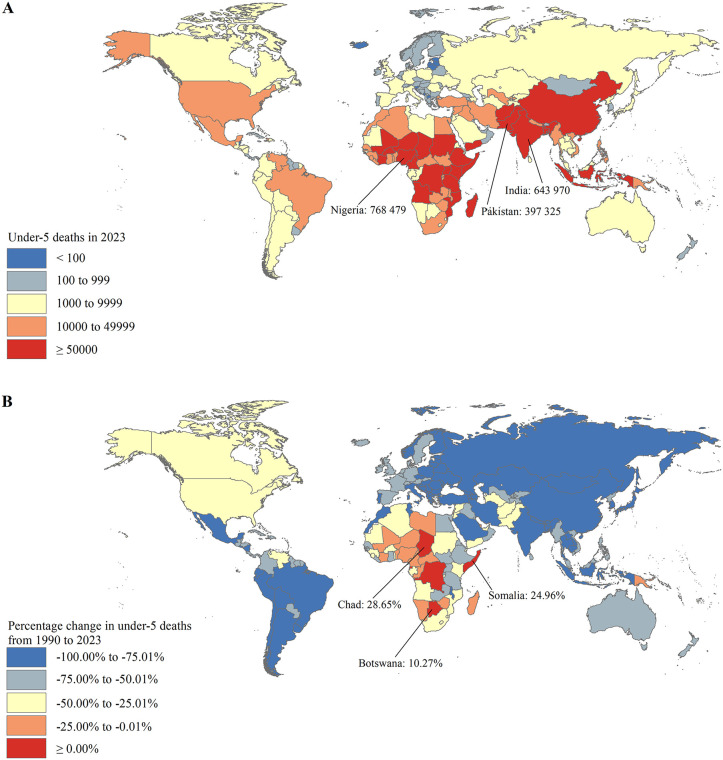
The under-5 deaths in 2023 (A) and the percentage change in under-5 deaths from 1990 to 2023 (B) at the national level.

Across the 200 countries and territories worldwide, the absolute number of under-5 deaths in Nigeria (0.77 (90% UI: 0.53, 1.13) million) and India (0.64 (90% UI: 0.55, 0.74) million) accounted for approximately one-third of the global under-5 deaths (4.78 (90% UI: 4.51, 5.33) million) in 2023 (Fig 3A and S2 Table). A total of 195 countries and territories experienced a decrease in the number of under-5 deaths from 1990 to 2023, with the most pronounced decrease in San Marino (−100.00%) and Montserrat (−100.00%), followed by Kosovo (−96.77%) (Fig 3B and S2 Table). Under-5 deaths increased from 1990 to 2023 in the remaining 5 countries, including Chad (28.65%), Somalia (24.96%), Botswana (10.27%), Democratic Republic of the Congo (3.92%), and Equatorial Guinea (3.15%) (Fig 3B and S2 Table).

**Fig 4:**
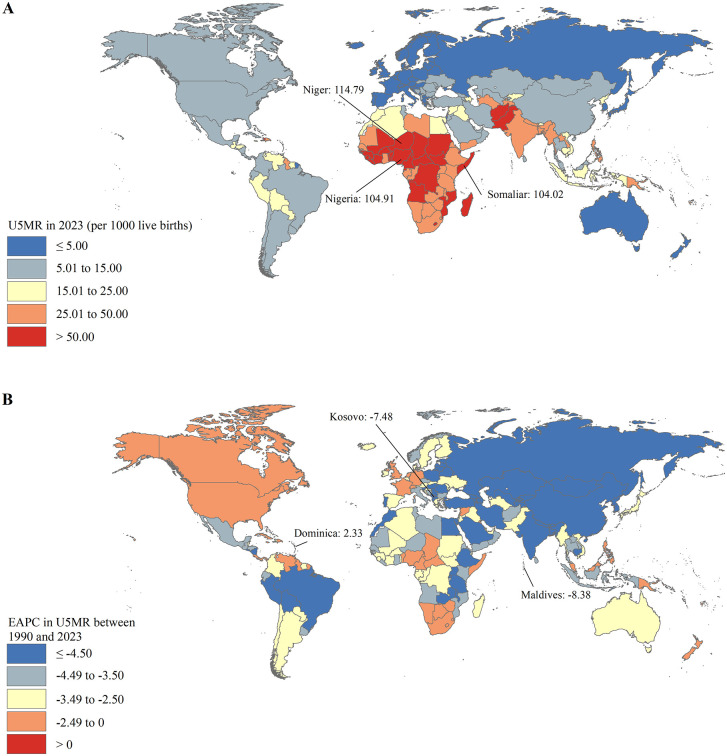
The U5MR in 2023 (A) and the EAPC in the U5MR between 1990 and 2023 (B) at the national level. EAPC: estimated annual percentage change; U5MR: under-5 mortality rate.

The U5MR varied considerably across the world, with the highest U5MR in 2023 in Niger (114.79 deaths per 1000 live births), followed by Nigeria (104.91 deaths per 1000 live births) and Somalia (104.02 deaths per 1000 live births) (Fig 4A and S2 Table). There were 133 countries and territories (67%) worldwide that achieved the SDG 3.2 U5MR target in 2023, such as Singapore, Australia, and China (Fig 4A and S2 Table). The remaining 67 countries and territories have U5MRs higher than 25.00 deaths per 1000 live births in 2023, 39 of which are 25.00 to 50.00 deaths per 1000 live births and 28 of which are higher than 50.00 deaths per 1000 live births (Fig 4A and S2 Table). The U5MRs were deemed to be in a decreasing trend in 192 countries and territories between 1990 and 2023, with the largest decrease in the Maldives (EAPC = −8.38; 95% CI: −8.64, −8.12), followed by Kosovo (EAPC = −7.48; 95% CI: −7.66, −7.29) and Estonia (EAPC = −7.06; 95% CI: −7.30, −6.82) (Fig 4B and S2 Table). The U5MRs were deemed to be in an increasing trend in Dominica (EAPC = 2.33; 95% CI: 2.15, 2.51) between 1990 and 2023 (Fig 4B and S2 Table). The U5MRs remained stable in Venezuela (Bolivarian Republic of), Syrian Arab Republic, Central African Republic, Grenada, Fiji, Niue, and Seychelles between 1990 and 2023 (Fig 4B and S2 Table).

**Fig 5:**
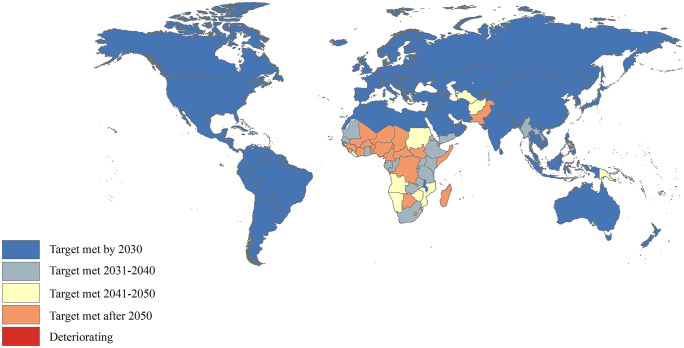
Years in which the U5MR reduction target is expected to be achieved. U5MR: under-5 mortality rate.

Among the 67 countries and territories with a U5MR higher than 25 deaths per 1000 live births in 2023, 58 could not achieve the SDG 3.2 U5MR target by 2030, and 9 countries, such as India, Nepal, and Bangladesh, were expected to reduce U5MR to at least as low as 25 deaths per 1000 live births by 2030 if the average rate of decline from 1990 to 2023 continues (Fig 5 and S3 Table). Almost three-quarters of the 58 that would not achieve the SDG 3.2 U5MR target by 2030 are in sub-Saharan Africa. 22 countries and territories, such as Myanmar, Kenya, and Yemen, were projected to meet the 2030 target in the subsequent decade (i.e., by 2040) (Fig 5 and S3 Table). Another 10 countries were projected to achieve the SDG 3.2 U5MR target between 2041 and 2050, such as Sudan, Angola, and Mozambique (Fig 5 and S3 Table). In particular, 25 countries and territories, such as Niger, Nigeria, and Pakistan, were projected to achieve the SDG 3.2 U5MR target after 2050 and the U5MR is expected to deteriorate in Dominica (Fig 5 and S3 Table).

## Discussion

This study used data on under-5 mortality compiled by the UN IGME to comprehensively assess the global, regional, and national trends in under-5 mortality between 1990 and 2023 and the progress towards SDG target 3.2. We found remarkable progress in improving child survival worldwide over the past three decades, with the global U5MR decreasing from 93.58 deaths per 1000 livebirths in 1990 to 36.72 deaths per 1000 livebirths in 2023. At the regional level, the U5MR decreased significantly in all SDG regions between 1990 and 2023, with nearly three-quarters (5/7) of the SDG regions already meeting the U5MR target by 2030 in 2023. At the national level, the U5MR decreased significantly in 192 countries and territories between 1990 and 2023 but increased significantly in Dominica and remained stable in the remaining 7 countries and territories during this period. Among the 200 countries and territories worldwide, 133 countries and territories have already met the U5MR target by 2030 in 2023, and 9 countries and territories would meet the target by 2030, 22 countries and territories by 2031–2040, 10 countries and territories by 2041–2050, and 25 countries and territories after 2050; however, the U5MR would be deteriorating in Dominica.

The global U5MR decreased by an average of 3.18% per year between 1990 and 2023, undeniably attributed to the intensive investments and targeted actions of the global health community to combat the main causes of child mortality with high-impact interventions, such as immunizations, access to nutrition and micronutrients, skilled attendants around birth and postnatal care, and expanded access to safe water, sanitation, and hygiene [25–29]. Despite substantial gains in reducing the global U5MR, progress has been insufficient to achieve SDG target 3.2 by 2030. If trends between 1990 and 2023 continue, SDG target 3.2 worldwide would be reached by 2035–5 years behind schedule. In addition, a large number of under-5 deaths remain, and children face widespread geographical disparities in their chances of survival. In 2023, more than four-fifths of the global under-5 deaths occurred in Sub-Saharan Africa and Central and Southern Asia, with U5MRs of 68.82 and 33.23 deaths per 1000 live births, respectively. The U5MR is projected to be lower than 25 deaths per 1000 livebirths in Sub-Saharan Africa by 2055 and in Central and Southern Asia by 2030. The other five SDG regions, Western Asia and Northern Africa, Eastern and South-Eastern Asia, Latin America and the Caribbean, Oceania, and North America and Europe, have already achieved the U5MR target in SDG 3.2 by 2023. Notably, despite recent gains in reducing U5M5 in Oceania, children’s health is at risk due to climate and environmental change [30]. Oceania countries are especially likely to be subjected to the effects of increases in ambient temperature, altered distribution of rainfall, ocean warming and sea level rise. Children are particularly vulnerable to climate and environmental change, and most predicted impacts will affect child health disproportionately [8]. Thus, reducing the global U5MR to 25 deaths per 1000 live births would primarily require an acceleration of progress for Sub-Saharan Africa. In Sub-Saharan Africa where most under-five deaths occur, nearly a third of all under-five deaths were caused by lower respiratory infections (pneumonia), malaria, and prematurity [6]. Notably, major infectious causes in sub-Saharan Africa had reductions in the past two decades, but infectious causes such as pneumonia, diarrhoea, malaria, and sepsis or meningitis remain important and should be a focus of child survival efforts going forward [31,32]. In South Asia, where neonatal mortality remains high relative to its under-five mortality level, the cause-of-death distribution is dominated by perinatal complications – for instance, one in four under-five deaths in this region is attributable to prematurity [6]. Importantly, as under-five survival improves overall, congenital anomalies have become responsible for a larger proportion of under-five deaths in Southern Asia [6]. A major focus of child survival programmes in this region has to be on neonatal causes.

Globally, 71% of the world’s countries and territories would achieve SDG target 3.2 with a U5MR of 25 or fewer deaths per 1000 livebirths before 2030, including 133 countries and territories by 2023 and 9 countries by 2030. The remaining 58 countries and territories would not meet the U5MR SDG target on time, specifically in Sub-Saharan Africa. Similarly, the UN IGME estimated that 59 countries are at risk of missing the SDG U5MR target based on U5MR between 1990 and 2022 [6]. In addition, we found that if trends between 1990 and 2023 were to continue, 58 countries and territories would not achieve the SDG 3.2 U5MR target by 2030, including 22 countries and territories projected to meet the target in 2030–2040, 10 in 2040–2050, 25 after 2050, and Dominica where the U5MR is deteriorating. Among the 58 countries and territories that are off track to achieve the SDG 3.2 U5MR target by 2030, 27 (46.55%) are classified as fragile and conflict-affected situations by the World Bank Group in 2025 [33]. Nearly 75% (43) of the countries and territories off track to meet the SDG 3.2 U5MR target by 2030 are in Sub-Saharan Africa, 90% of which were classified as low- or lower-middle-income countries according to the World Bank’s country classification [34]. This means that there is still work to be done in improving child survival in these countries and territories to accelerate progress to achieve the U5MR SDG target. The world must take action to save the lives of children who remain vulnerable, marginalized and, in many cases, overlooked by decision-makers. Globally, infectious causes, preterm birth complications, lower respiratory infections, intrapartum-related events, and diarrhea remain the leading causes of death for children under 5 years of age [31,32]. Therefore, improving access to basic lifesaving interventions such as skilled delivery at birth, postnatal care, breastfeeding and adequate nutrition, vaccinations, and treatment for common childhood diseases could save many young lives in these countries. Over the past three decades, China has made substantial progress in promoting child health, with a notable reduction in mortality in children younger than 5 years, and has now achieved SDG 3.2 U5MR target. From 2016 to 2022, under-5 deaths caused by acute respiratory infections, preterm birth complications, intrapartum-related events, and congenital malformations declined rapidly in China, altogether accounting for 76.5% of the total decrease in under-5 deaths [35]. The reduction in mortality from acute respiratory infections and diarrhoea could potentially be attributed to improvements in socioeconomic status, child nutrition, water and sanitation, pneumococcal vaccine coverage, and the National Essential Public Health Programmes, which continue to advance and has increased access to comprehensive management of childhood diseases and health management for children aged 0–6 years [36–39]. Medical progress and political will have also contributed to the continued decline in deaths due to preterm birth complications and congenital malformations. For instance, a previous study found that the survival rate after discharge from hospital for extremely preterm infants with a gestational age of 24–27 weeks increased in China from 2010 to 2019 [40]. National strategies, policies, and programmes, such as the health-care working specification for preterm infants, the integrated prevention and treatment of birth defects (e.g., folic acid supplementation and improved insurance coverage for surgeries addressing congenital heart diseases), and the National Action Plan on Disability Prevention (2016–20), have also contributed to the decline in deaths from both causes [35,41–44]. The steady decrease in deaths due to intrapartum-related events is probably associated with progress in neonatal resuscitation techniques and deliveries [37,45,46]. Notably, one country, Dominica, could not achieve SDG target 3.2 if the average rate of increase from 1990 to 2023 continues. One previous study indicated that intrapartum complications, as a cause of death in children under 5 years of age, more than tripled between 2000 and 2016 in Dominica [47]. In 2016, more than 35% of all deaths in children under 5 years of age in Dominica were caused by intrapartum complications [47]. In no other country in the world do intrapartum complications constitute more than 13% of deaths in children under 5 years of age [47]. Therefore, addressing intrapartum complications would be an effective way to reduce the U5MR in Dominica.

With 2030 approaching quickly, progress must be prioritized and accelerated to ensure every child’s right to survive is upheld. Findings of this study emphasize that investments at local, subnational and national levels must be made to ensure proven interventions are available and accessible in every community, especially in Sub-Saharan Africa, where children are most at risk. The effective measures to save children’s lives include scaling up high-impact interventions: skilled health personnel at birth, care for small and sick newborns, antenatal and postnatal care, preventive services such as vaccination, improved access to diagnosis and treatment of key causes of childhood illness and death, and efforts to reduce mortality risk factors including malnutrition [6]. To ensure the survival of children, priority must be given not only to coverage, but also to equity and quality. Meanwhile, our findings provide evidence-based guidance for optimizing donor prioritization and resource allocation, directing critical funding to high-burden regions where child survival needs are most acute, such as Sub-Saharan Africa. Beyond resource mobilization, strengthening national data systems is essential to establish robust monitoring and accountability mechanisms, enabling real-time tracking of progress toward SDG 3.2 and facilitating timely course correction to keep efforts aligned with 2030 goals. According to the latest report of Levels & Trends in Child Mortality Report by UN IGME, data are least available precisely in the regions where children face the highest risks [6]. Data availability declines by income group, in countries in fragile and conflict-affected situations, and among countries at risk of missing the SDG targets for child mortality. Data and statistical systems – particularly those that record the demographic components needed for mortality estimation (i.e., births, deaths and population) – must be improved to track and monitor survival by age, and direct resources towards the most marginalized children. In addition, integrating data collected across the life-course is critical, along with efforts to bolster the completeness, timeliness and quality of data collected by various local and national data producers.

### Strengths and limitations

The current study comprehensively assessed the global, regional, and national trends in U5MRs between 1990 and 2023 and their progress towards SDG target 3.2 using data compiled by the UN IGME. The 33-year span provides a rich historical context for assessing progress and forecasting future trends. Furthermore, the multi-level disaggregation at the global, regional, and national levels enhances the policy relevance and analytical granularity of the study. However, several limitations should be noted. First, the UN IGME estimates are the most comprehensive, transparent, and up-to-date information on child survival, providing the basis for assessing progress towards survival goals and for evidence-based policies. However, despite the expansion of data availability and advances in analytical methods, the lack of timely, high-quality data on child mortality for many countries is one of the limitations of this study. Moreover, mortality estimates may be less reliable in conflict-affected or data-poor contexts. Second, the EAPCs in U5MRs and the relative change in under-5 deaths were used to assess their long-term trends from 1990 to 2023, which might mask the recent short-term trends that reflected the effectiveness of recent prevention interventions. Third, it should be emphasized that projections of U5MR beyond 2023 are scenario-based estimates rather than definitive predictions, and they rely on the EAPCs in U5MRs between 1990 and 2023 and the U5MR in 2023. These extrapolations carry considerable uncertainty, as future U5MR trajectories could be fundamentally disrupted by unforeseen global shocks such as pandemics, inter- or intra-state conflicts, and other large-scale systemic disruptions. Fourth, although this study aims to provide an exhaustive description of trends in U5MR at the global, regional, and national levels and their progress towards SDG target 3.2, it does not directly address the underlying causes of death for children under 5 years of age or specific interventions that might be directly associated with under-5 deaths. Fifth, the lack of subnational analysis constitutes another limitation – national averages may obscure substantial within-country disparities, particularly in large, demographically diverse countries. Finally, the UN IGME estimates do not include any adjustment in the years 2020, 2021, 2022, or 2023 for COVID-19-related mortality as the evidence is insufficient to support an adjustment at this time, which may result in potential biases in projections.

## Conclusions

In summary, among the 200 countries and territories worldwide, 133 have already met SDG target 3.2, and 9 are expected to do so by 2030, 22 would meet it between 2031 and 2040, 10 would meet it between 2041 and 2050, and 25 would meet it after 2050, if the average rate of decline from 1990 to 2023 continues; however, the U5MR would deteriorate in Dominica. Investments of skilled health personnel at birth, care for small and sick newborns, antenatal and postnatal care, preventive services such as vaccination, improved access to diagnosis and treatment of key causes of childhood illness and death, and efforts to reduce mortality risk factors including malnutrition at local, subnational and national levels must be made to ensure proven interventions are available and accessible in every community, especially in Sub-Saharan Africa, where children are most at risk.

## Supporting information

S1 Table Years in which U5MR reduction target is expected to be achieved. U5MR: under-5 mortality rate. (PDF)

S2 Table Under-5 deaths and U5MRs in 1990 and 2023 and their trends from 1990 to 2023 at national level. CI: confidence interval; EAPC: estimated annual percentage change; U5MR: under-5 mortality rate; UI: uncertainty interval.
(PDF)

S3 Table Years in which U5MR reduction target is expected to be achieved at the national level. U5MR: under-5 mortality rate.
(PDF)
