# Supplementary material for: Tracking under-five mortality from 1990 to 2023: Global, regional, and national trends, inequities, and projections toward achieving SDG Target 3.2 by 2030
Source: PLoS One. 2026 Apr 1;21(4):e0343745. doi: 10.1371/journal.pone.0343745 (PMC13042728; doi:10.1371/journal.pone.0343745)
Supplement: S1 Table — (PDF) [file pone.0343745.s001.pdf]

S1 Table. Years in which U5MR reduction target is expected to be achieved

Characteristics	Year
Global	2035
Sex	
Male	2038
Female	2033
UNICEF region	
Sub-Saharan Africa	2055
Western Asia and Northern Africa	2023
Central and Southern Asia	2030
Eastern and South-Eastern Asia	2023
Latin America and Caribbean	2023
Oceania	2023
North America and Europe	2023

U5MR: under-5 mortality rate.
