# Supplementary material for: Tracking under-five mortality from 1990 to 2023: Global, regional, and national trends, inequities, and projections toward achieving SDG Target 3.2 by 2030
Source: PLoS One. 2026 Apr 1;21(4):e0343745. doi: 10.1371/journal.pone.0343745 (PMC13042728; doi:10.1371/journal.pone.0343745)
Supplement: S2 Table — CI: confidence interval; EAPC: estimated annual percentage change; U5MR: under-5 mortality rate; UI: uncertainty interval. (PDF) [file pone.0343745.s002.pdf]

S2 Table. Under-5 deaths and U5MRs in 1990 and 2023 and their trends from 1990 to 2023 at national level

| Region | 1990 | | 2023 | | 1990-2023 | |
|---|---|---|---|---|---|---|
| | Deaths<br>No. (90% UI) | Mortality rate per 1000 live births<br>No. (90% UI) | Deaths<br>No. (90% UI) | Mortality rate per 1000 live births<br>No. (90% UI) | Percentage change in deaths<br>No. (%) | EAPC in mortality rate<br>No. (95% CI) |
| Low-income country | | | | | | |
| Afghanistan | 109143 (99940, 119076) | 180.66 (165.05, 197.58) | 80507 (61618, 105235) | 55.51 (42.42, 72.70) | -26.24 | -3.62 (-3.69, -3.56) |
| Burkina Faso | 81780 (76511, 87452) | 198.28 (185.48, 212.12) | 55356 (35879, 85811) | 77.27 (50.06, 119.65) | -32.31 | -3.14 (-3.33, -2.94) |
| Burundi | 43289 (38938, 48096) | 169.84 (152.73, 188.75) | 22339 (13037, 37982) | 49.21 (28.68, 83.79) | -48.4 | -4.36 (-4.66, -4.05) |
| Central African Republic | 22973 (20752, 25356) | 177.49 (160.13, 196.18) | 20141 (15167, 26527) | 92.20 (68.59, 123.48) | -12.33 | -0.39 (-1.48, 0.71) |
| Chad | 61360 (56859, 66149) | 211.62 (195.98, 228.42) | 78937 (53407, 116133) | 101.13 (68.37, 148.61) | 28.65 | -2.22 (-2.33, -2.11) |
| Democratic Republic of the Congo | 294908 (265985, 326846) | 185.31 (167.05, 205.66) | 306481 (176412, 525984) | 73.19 (42.08, 125.55) | 3.92 | -2.96 (-3.10, -2.83) |
| Eritrea | 12976 (11835, 14179) | 153.59 (139.94, 168.09) | 3439 (1881, 6378) | 35.38 (19.33, 65.72) | -73.5 | -4.35 (-4.56, -4.14) |
| Gambia | 7814 (7105, 8626) | 166.10 (150.77, 183.69) | 3556 (2613, 4806) | 44.08 (32.38, 59.59) | -54.49 | -2.98 (-3.16, -2.80) |
| Guinea-Bissau | 9650 (8651, 10764) | 222.33 (198.86, 248.54) | 4404 (2494, 7589) | 69.30 (39.17, 119.85) | -54.36 | -2.66 (-2.83, -2.49) |
| Democratic People's Republic of Korea | 17165 (13453, 21955) | 40.74 (31.91, 52.16) | 6196 (4854, 7929) | 18.00 (14.09, 23.04) | -63.9 | -4.91 (-6.27, -3.52) |
| Liberia | 30241 (27783, 32975) | 267.77 (246.06, 291.91) | 12129 (8696, 16936) | 72.88 (52.25, 101.79) | -59.89 | -4.43 (-4.70, -4.16) |
| Madagascar | 77893 (72705, 83456) | 155.48 (145.11, 166.73) | 63429 (50488, 80909) | 64.77 (51.55, 82.58) | -18.57 | -2.86 (-3.16, -2.55) |
| Malawi | 103659 (97910, 109961) | 242.78 (229.30, 257.65) | 24893 (14243, 42341) | 38.34 (21.91, 65.28) | -75.99 | -6.02 (-6.24, -5.79) |
| Mali | 96772 (91147, 102869) | 231.03 (217.44, 245.80) | 83597 (62883, 111276) | 91.30 (68.69, 121.43) | -13.61 | -2.92 (-2.99, -2.85) |
| Mozambique | 142508 (131296, 154539) | 232.95 (214.31, 252.89) | 75675 (61530, 93321) | 61.65 (50.11, 76.06) | -46.9 | -4.43 (-4.61, -4.25) |
| Niger | 141955 (132923, 151516) | 331.67 (310.44, 354.12) | 119782 (91890, 154726) | 114.79 (88.13, 148.00) | -15.62 | -3.50 (-3.79, -3.21) |
| Rwanda | 48631 (45652, 51765) | 151.35 (142.11, 161.08) | 15705 (12145, 20296) | 40.01 (30.96, 51.68) | -67.71 | -6.17 (-6.95, -5.37) |
| Sierra Leone | 47001 (43378, 50730) | 259.32 (239.14, 280.31) | 23993 (18797, 31046) | 94.28 (73.93, 122.00) | -48.95 | -3.21 (-3.41, -3.02) |
| Somalia | 63120 (51964, 77299) | 179.96 (148.09, 220.73) | 78874 (36907, 169412) | 104.02 (48.32, 225.93) | 24.96 | -1.97 (-2.88, -1.06) |
| South Sudan | 68560 (58108, 76617) | 302.76 (250.49, 356.18) | 31441 (9146, 81204) | 98.73 (28.63, 255.74) | -54.14 | -3.13 (-4.23, -2.01) |
| Sudan | 122944 (113887, 132829) | 131.82 (122.03, 142.50) | 82559 (53674, 125462) | 50.15 (32.54, 76.32) | -32.85 | -3.00 (-3.12, -2.88) |
| Syrian Arab Republic | 17147 (15569, 18937) | 36.99 (33.57, 40.87) | 10134 (4962, 15308) | 20.56 (10.05, 31.08) | -40.9 | -0.52 (-1.35, 0.33) |
| Togo | 23543 (21871, 25284) | 147.18 (136.62, 158.21) | 16559 (11799, 23140) | 58.29 (41.53, 81.33) | -29.66 | -2.88 (-2.95, -2.80) |
| Uganda | 153620 (144629, 163272) | 182.80 (172.02, 194.34) | 65043 (42358, 98115) | 38.81 (25.25, 58.53) | -57.66 | -5.13 (-5.40, -4.86) |
| Yemen | 84567 (79321, 90231) | 125.03 (117.19, 133.47) | 53148 (42445, 67573) | 39.25 (31.32, 49.97) | -37.15 | -3.73 (-3.93, -3.54) |
| Middle-income country | | | | | | |
| Albania | 3430 (3057, 3855) | 40.77 (36.32, 45.86) | 273 (239, 311) | 9.37 (8.21, 10.70) | -92.04 | -5.20 (-5.55, -4.84) |

| | | | | | | |
|---|---|---|---|---|---|---|
| Algeria | 39818 (36904, 42776) | 51.70 (47.91, 55.54) | 20295 (18314, 22500) | 22.00 (19.85, 24.39) | -49.03 | -2.92 (-3.06, -2.78) |
| Angola | 122572 (108893, 137643) | 222.70 (197.43, 250.43) | 85579 (35971, 176138) | 64.01 (26.82, 131.97) | -30.18 | -4.29 (-4.62, -3.96) |
| Argentina | 20485 (20106, 20874) | 28.79 (28.25, 29.33) | 4920 (4331, 5589) | 9.65 (8.49, 10.96) | -75.98 | -3.37 (-3.45, -3.28) |
| Armenia | 4159 (3769, 4581) | 49.01 (44.37, 54.02) | 348 (278, 439) | 10.04 (8.03, 12.68) | -91.63 | -4.75 (-4.76, -4.73) |
| Azerbaijan | 19772 (17896, 21868) | 94.83 (85.76, 105.00) | 2443 (1607, 3738) | 18.56 (12.21, 28.40) | -87.64 | -5.49 (-5.73, -5.25) |
| Bangladesh | 569897 (551386, 588727) | 145.97 (141.19, 150.80) | 105531 (100884, 110840) | 30.56 (29.21, 32.09) | -81.48 | -4.99 (-5.15, -4.83) |
| Belarus | 2238 (2190, 2286) | 15.20 (14.88, 15.53) | 177 (141, 221) | 2.44 (1.94, 3.04) | -92.09 | -6.20 (-6.57, -5.83) |
| Belize | 261 (230, 297) | 39.26 (34.57, 44.72) | 92 (82, 102) | 12.74 (11.43, 14.16) | -64.75 | -3.23 (-3.36, -3.11) |
| Benin | 39619 (37316, 42163) | 172.21 (162.08, 183.36) | 36395 (27819, 47930) | 77.90 (59.56, 102.45) | -8.14 | -2.29 (-2.32, -2.27) |
| Bhutan | 2815 (2446, 3286) | 128.20 (111.16, 149.88) | 229 (125, 416) | 23.06 (12.55, 42.05) | -91.87 | -5.47 (-5.63, -5.31) |
| Bolivia (Plurinational State of) | 30477 (28877, 32216) | 121.70 (115.23, 128.72) | 5993 (4007, 8947) | 23.10 (15.44, 34.51) | -80.34 | -5.22 (-5.32, -5.12) |
| Bosnia and Herzegovina | 1263 (1232, 1296) | 18.18 (17.73, 18.66) | 151 (138, 165) | 6.04 (5.54, 6.59) | -88.04 | -4.21 (-5.47, -2.94) |
| Botswana | 2190 (1829, 2636) | 50.07 (41.80, 60.31) | 2415 (912, 6302) | 39.57 (14.89, 104.37) | 10.27 | -1.54 (-2.10, -0.97) |
| Brazil | 234441 (218780, 251129) | 63.08 (58.83, 67.59) | 38010 (33450, 43045) | 14.44 (12.71, 16.36) | -83.79 | -4.73 (-5.09, -4.36) |
| Cabo Verde | 839 (810, 870) | 56.38 (54.39, 58.45) | 75 (66, 85) | 11.59 (10.24, 13.11) | -91.06 | -4.80 (-5.09, -4.51) |
| Cambodia | 38303 (35417, 41471) | 116.26 (107.42, 125.91) | 8329 (3969, 17870) | 22.90 (10.89, 49.23) | -78.25 | -5.85 (-6.27, -5.44) |
| Cameroon | 64641 (60146, 69572) | 136.65 (127.08, 147.14) | 63046 (46857, 84809) | 67.24 (49.99, 90.41) | -2.47 | -2.46 (-2.77, -2.15) |
| China | 1463332 (1353099, 1593014) | 53.66 (49.60, 58.45) | 62190 (56139, 69280) | 6.19 (5.59, 6.90) | -95.75 | -6.95 (-7.21, -6.70) |
| Colombia | 30609 (28518, 32792) | 35.69 (33.24, 38.24) | 8500 (5397, 13338) | 12.03 (7.63, 18.89) | -72.23 | -3.21 (-3.24, -3.19) |
| Comoros | 2287 (2033, 2556) | 120.92 (107.36, 135.47) | 961 (725, 1272) | 39.82 (30.01, 52.77) | -57.98 | -3.30 (-3.44, -3.15) |
| Congo | 8139 (7153, 9232) | 91.48 (80.43, 103.82) | 7520 (4005, 14169) | 40.51 (21.54, 76.39) | -7.61 | -3.21 (-3.69, -2.73) |
| Côte d'Ivoire | 82205 (76684, 88325) | 153.05 (142.62, 164.57) | 65790 (53648, 80605) | 67.13 (54.72, 82.30) | -19.97 | -2.74 (-2.91, -2.57) |
| Cuba | 2492 (2217, 2809) | 13.43 (11.95, 15.15) | 806 (659, 1007) | 8.27 (6.76, 10.34) | -67.66 | -1.89 (-2.42, -1.34) |
| Djibouti | 2300 (1968, 2665) | 116.31 (99.38, 135.02) | 1198 (638, 2232) | 50.42 (26.76, 94.44) | -47.91 | -2.56 (-2.67, -2.44) |
| Dominica | 34 (32, 36) | 18.52 (17.44, 19.69) | 26 (23, 30) | 35.47 (30.80, 41.05) | -23.53 | 2.33 (2.15, 2.51) |
| Dominican Republic | 12858 (12062, 13717) | 59.47 (55.76, 63.46) | 6396 (4513, 9100) | 31.41 (22.14, 44.75) | -50.26 | -1.54 (-1.81, -1.28) |
| Ecuador | 16563 (14895, 18421) | 53.88 (48.43, 59.95) | 3581 (3228, 3952) | 13.05 (11.77, 14.41) | -78.38 | -4.37 (-4.59, -4.15) |
| Egypt | 166615 (158587, 175203) | 85.68 (81.51, 90.13) | 41799 (26161, 67525) | 17.53 (10.96, 28.35) | -74.91 | -4.74 (-4.92, -4.56) |
| El Salvador | 10687 (9777, 11677) | 59.39 (54.30, 64.95) | 1041 (639, 1755) | 10.43 (6.40, 17.60) | -90.26 | -5.23 (-5.36, -5.09) |
| Equatorial Guinea | 3685 (3261, 4122) | 178.72 (157.89, 200.16) | 3801 (2063, 6995) | 70.62 (38.23, 130.23) | 3.15 | -2.97 (-3.12, -2.82) |
| Eswatini | 2332 (2070, 2628) | 67.53 (59.88, 76.20) | 1333 (902, 2012) | 45.01 (30.39, 68.17) | -42.84 | -1.89 (-2.76, -1.01) |
| Fiji | 660 (570, 761) | 28.89 (24.94, 33.35) | 488 (412, 583) | 29.14 (24.58, 34.79) | -26.06 | -0.06 (-0.42, 0.30) |
| Gabon | 3044 (2661, 3458) | 84.86 (74.11, 96.52) | 2273 (1630, 3275) | 33.21 (23.81, 47.90) | -25.33 | -2.98 (-3.16, -2.80) |

| | | | | | | |
|---|---|---|---|---|---|---|
| Georgia | 4914 (4395, 5505) | 47.49 (42.45, 53.21) | 409 (341, 497) | 9.17 (7.64, 11.13) | -91.68 | -6.07 (-6.50, -5.63) |
| Ghana | 77058 (72909, 81510) | 127.48 (120.58, 134.85) | 32579 (25963, 40841) | 37.06 (29.52, 46.47) | -57.72 | -3.83 (-3.99, -3.67) |
| Grenada | 62 (58, 67) | 22.38 (20.89, 24.07) | 25 (20, 32) | 18.27 (14.32, 23.21) | -59.68 | -0.10 (-0.50, 0.30) |
| Guatemala | 28033 (26264, 29820) | 80.37 (75.25, 85.54) | 8055 (5544, 11797) | 21.36 (14.69, 31.30) | -71.27 | -3.95 (-3.99, -3.91) |
| Guinea | 64662 (60491, 69211) | 233.13 (217.77, 249.76) | 45350 (35004, 59320) | 95.03 (73.39, 124.21) | -29.87 | -2.66 (-2.83, -2.49) |
| Haiti | 36735 (34242, 39383) | 144.92 (134.97, 155.53) | 14233 (9416, 21660) | 55.12 (36.42, 83.98) | -61.25 | -2.76 (-3.09, -2.42) |
| Honduras | 10847 (10031, 11697) | 58.13 (53.74, 62.74) | 3605 (2602, 5113) | 15.51 (11.19, 22.01) | -66.77 | -4.05 (-4.34, -3.77) |
| India | 3508138 (3388812, 3630467) | 126.52 (122.17, 130.97) | 643970 (553749, 740518) | 27.75 (23.85, 31.91) | -81.64 | -4.61 (-4.80, -4.42) |
| Indonesia | 396615 (378356, 415372) | 83.87 (79.97, 87.86) | 92839 (68544, 125802) | 20.62 (15.22, 27.96) | -76.59 | -4.21 (-4.31, -4.11) |
| Iran (Islamic Republic of) | 113465 (104114, 123807) | 57.57 (52.79, 62.86) | 13987 (7659, 25153) | 11.77 (6.44, 21.19) | -87.67 | -5.00 (-5.16, -4.84) |
| Iraq | 54331 (49530, 59606) | 79.74 (72.65, 87.51) | 25947 (18136, 36826) | 22.62 (15.79, 32.14) | -52.24 | -2.89 (-3.11, -2.67) |
| Jamaica | 1726 (1439, 2078) | 28.03 (23.35, 33.77) | 643 (597, 692) | 19.33 (17.95, 20.79) | -62.75 | -1.06 (-1.31, -0.81) |
| Jordan | 4500 (4200, 4800) | 35.44 (33.07, 37.82) | 3108 (2318, 4330) | 13.21 (9.85, 18.42) | -30.93 | -2.94 (-2.97, -2.90) |
| Kazakhstan | 20870 (18956, 22995) | 51.58 (46.82, 56.87) | 4019 (3885, 4158) | 9.65 (9.33, 9.98) | -80.74 | -6.19 (-6.65, -5.73) |
| Kenya | 99132 (93008, 105869) | 101.45 (95.16, 108.39) | 59036 (49351, 70655) | 39.90 (33.33, 47.79) | -40.45 | -3.60 (-3.89, -3.31) |
| Kiribati | 250 (221, 282) | 91.28 (80.63, 103.03) | 188 (118, 293) | 55.14 (34.46, 85.67) | -24.8 | -1.23 (-1.46, -1.00) |
| Kosovo (UNSCR 1244) | 5891 (4556, 7797) | 103.83 (79.92, 138.58) | 190 (155, 233) | 9.11 (7.42, 11.17) | -96.77 | -7.48 (-7.66, -7.29) |
| Kyrgyzstan | 9194 (8165, 10339) | 65.17 (57.82, 73.38) | 2572 (2445, 2722) | 16.98 (16.15, 17.97) | -72.03 | -4.69 (-4.89, -4.48) |
| Lao People's Democratic Republic | 27013 (24782, 29476) | 154.14 (141.27, 168.42) | 6350 (4236, 9438) | 39.00 (25.96, 58.08) | -76.49 | -4.25 (-4.33, -4.18) |
| Lebanon | 3166 (2827, 3550) | 31.77 (28.35, 35.64) | 1723 (1548, 1918) | 18.31 (16.46, 20.39) | -45.58 | -2.31 (-2.95, -1.66) |
| Lesotho | 5357 (4868, 5913) | 84.71 (76.89, 93.64) | 3260 (2391, 4310) | 58.93 (43.12, 78.06) | -39.15 | -1.33 (-1.88, -0.77) |
| Libya | 5134 (4360, 6016) | 41.91 (35.55, 49.16) | 3974 (2196, 7135) | 30.78 (17.05, 54.98) | -22.59 | -3.82 (-4.49, -3.14) |
| Malaysia | 7645 (7445, 7839) | 16.61 (16.17, 17.03) | 3523 (3176, 3904) | 8.08 (7.29, 8.95) | -53.92 | -2.05 (-2.48, -1.61) |
| Maldives | 786 (717, 862) | 85.66 (78.10, 94.08) | 34 (28, 41) | 5.71 (4.68, 6.94) | -95.67 | -8.38 (-8.64, -8.12) |
| Marshall Islands | 90 (76, 105) | 47.59 (40.52, 55.68) | 25 (14, 43) | 28.18 (15.98, 49.11) | -72.22 | -1.22 (-1.41, -1.04) |
| Mauritania | 8972 (8148, 9848) | 115.40 (104.76, 126.74) | 6402 (4566, 8989) | 37.80 (26.94, 53.14) | -28.64 | -3.79 (-4.00, -3.58) |
| Mauritius | 504 (484, 525) | 23.17 (22.26, 24.12) | 183 (166, 202) | 15.17 (13.75, 16.73) | -63.69 | -1.41 (-1.73, -1.09) |
| Mexico | 109619 (101714, 117751) | 45.31 (42.02, 48.69) | 25584 (20414, 31811) | 12.46 (9.94, 15.49) | -76.66 | -3.78 (-3.90, -3.66) |
| Micronesia (Federated States of) | 174 (144, 208) | 49.31 (40.86, 59.20) | 58 (26, 127) | 23.08 (10.40, 50.91) | -66.67 | -2.10 (-2.22, -1.98) |
| Republic of Moldova | 2883 (2487, 3342) | 33.49 (28.87, 38.84) | 482 (369, 654) | 14.67 (11.24, 19.94) | -83.28 | -3.47 (-3.91, -3.03) |
| Mongolia | 7846 (7220, 8534) | 106.49 (97.93, 115.98) | 915 (879, 952) | 13.64 (13.10, 14.19) | -88.34 | -6.73 (-6.96, -6.50) |
| Montenegro | 160 (152, 168) | 16.50 (15.71, 17.35) | 18 (15, 22) | 2.58 (2.14, 3.13) | -88.75 | -6.17 (-6.69, -5.65) |
| Morocco | 58589 (54433, 62944) | 80.78 (75.01, 86.82) | 10483 (6875, 15603) | 16.58 (10.86, 24.69) | -82.11 | -4.88 (-4.97, -4.79) |

| | | | | | | |
|---|---|---|---|---|---|---|
| Myanmar | 123627 (112841, 135849) | 115.10 (104.94, 126.62) | 35008 (21282, 55399) | 38.74 (23.51, 61.42) | -71.68 | -3.35 (-3.63, -3.08) |
| Namibia | 3734 (3372, 4134) | 73.90 (66.69, 81.86) | 3085 (2508, 4145) | 40.65 (33.02, 54.69) | -17.38 | -1.87 (-2.18, -1.56) |
| Nepal | 99751 (93837, 106034) | 138.90 (130.56, 147.80) | 15282 (12296, 18931) | 26.47 (21.29, 32.80) | -84.68 | -4.91 (-4.99, -4.82) |
| Nicaragua | 10004 (9264, 10807) | 66.21 (61.28, 71.57) | 1780 (1648, 2034) | 13.43 (12.43, 15.35) | -82.21 | -4.81 (-4.95, -4.68) |
| Nigeria | 864182 (807605, 922934) | 209.77 (196.01, 224.17) | 768479 (533175, 1128658) | 104.91 (72.74, 153.95) | -11.07 | -2.24 (-2.34, -2.14) |
| North Macedonia | 1393 (1355, 1431) | 36.97 (35.97, 37.98) | 59 (49, 70) | 3.26 (2.72, 3.87) | -95.76 | -5.42 (-6.02, -4.83) |
| Pakistan | 670014 (645431, 695650) | 139.81 (134.62, 145.24) | 397325 (324825, 487344) | 58.46 (47.76, 71.73) | -40.7 | -2.53 (-2.59, -2.48) |
| Papua New Guinea | 11784 (10688, 12929) | 84.66 (76.72, 92.98) | 10262 (6546, 15937) | 40.31 (25.68, 62.69) | -12.92 | -2.16 (-2.27, -2.06) |
| Paraguay | 6236 (5589, 6941) | 45.54 (40.79, 50.71) | 2332 (1117, 4961) | 17.02 (8.14, 36.26) | -62.6 | -2.82 (-2.88, -2.77) |
| Peru | 54402 (51671, 57348) | 80.41 (76.35, 84.81) | 8536 (6538, 11369) | 15.81 (12.10, 21.06) | -84.31 | -5.09 (-5.56, -4.61) |
| Philippines | 118817 (111037, 127147) | 56.01 (52.33, 59.94) | 49616 (37910, 66290) | 26.88 (20.53, 35.93) | -58.24 | -2.01 (-2.25, -1.77) |
| Samoa | 174 (152, 198) | 29.80 (26.04, 34.00) | 88 (58, 134) | 15.70 (10.38, 23.93) | -49.43 | -1.51 (-1.78, -1.23) |
| Sao Tome and Principe | 521 (460, 589) | 108.13 (95.55, 122.45) | 89 (49, 163) | 13.94 (7.70, 25.60) | -82.92 | -6.91 (-7.27, -6.55) |
| Senegal | 42784 (40661, 44995) | 137.43 (130.58, 144.53) | 19997 (16331, 24672) | 38.52 (31.46, 47.53) | -53.26 | -4.42 (-4.76, -4.08) |
| Serbia | 2630 (2570, 2688) | 28.46 (27.81, 29.09) | 319 (297, 342) | 5.23 (4.87, 5.62) | -87.87 | -4.99 (-5.40, -4.58) |
| Solomon Islands | 472 (413, 538) | 38.20 (33.39, 43.61) | 437 (295, 657) | 20.55 (13.86, 30.89) | -7.42 | -1.62 (-1.70, -1.55) |
| South Africa | 68817 (60567, 78622) | 58.17 (51.14, 66.54) | 40976 (36632, 45877) | 34.65 (30.99, 38.79) | -40.46 | -2.26 (-2.97, -1.53) |
| Sri Lanka | 7779 (7623, 7938) | 23.30 (22.83, 23.78) | 1989 (1728, 2284) | 6.11 (5.31, 7.01) | -74.43 | -4.13 (-4.69, -3.56) |
| Saint Lucia | 83 (79, 87) | 20.89 (19.93, 21.93) | 32 (27, 37) | 15.55 (13.23, 18.29) | -61.45 | -0.68 (-0.75, -0.61) |
| Saint Vincent and the Grenadines | 62 (58, 66) | 23.77 (22.18, 25.46) | 13 (10, 17) | 10.64 (8.14, 13.87) | -79.03 | -2.07 (-2.53, -1.61) |
| Suriname | 566 (481, 663) | 44.95 (38.21, 52.74) | 176 (107, 301) | 16.23 (9.84, 27.82) | -68.9 | -2.94 (-3.03, -2.86) |
| Tajikistan | 22381 (20386, 24543) | 99.22 (90.31, 108.85) | 7457 (5593, 10009) | 27.32 (20.49, 36.69) | -66.68 | -5.06 (-5.47, -4.65) |
| United Republic of Tanzania | 180806 (170292, 191927) | 166.87 (157.07, 177.17) | 89057 (69823, 112483) | 38.86 (30.45, 49.09) | -50.74 | -4.82 (-5.01, -4.63) |
| Thailand | 39677 (37010, 42292) | 36.94 (34.45, 39.39) | 5526 (4945, 6912) | 9.21 (8.24, 11.53) | -86.07 | -4.09 (-4.23, -3.95) |
| Timor-Leste | 7027 (6334, 7785) | 225.41 (202.56, 250.62) | 1526 (1065, 2248) | 49.95 (34.80, 73.66) | -78.28 | -4.91 (-5.39, -4.44) |
| Tonga | 73 (61, 88) | 22.15 (18.55, 26.45) | 24 (16, 36) | 9.93 (6.49, 14.92) | -67.12 | -2.32 (-2.37, -2.27) |
| Tunisia | 12067 (10575, 13782) | 54.96 (48.15, 62.79) | 2221 (2109, 2337) | 12.91 (12.25, 13.58) | -81.59 | -3.93 (-4.34, -3.52) |
| Turkey | 106645 (99314, 114295) | 73.82 (68.71, 79.17) | 14251 (12245, 16884) | 12.79 (10.99, 15.15) | -86.64 | -6.22 (-6.53, -5.92) |
| Turkmenistan | 10364 (9121, 11866) | 79.15 (69.58, 90.78) | 6483 (3968, 10573) | 39.95 (24.42, 65.22) | -37.45 | -2.50 (-2.81, -2.19) |
| Tuvalu | 16 (13, 19) | 53.72 (45.18, 63.87) | 5 (2, 9) | 19.89 (10.59, 36.43) | -68.75 | -3.10 (-3.20, -2.99) |
| Ukraine | 13471 (12136, 15271) | 19.24 (17.33, 21.82) | 1823 (1654, 2041) | 8.07 (7.33, 9.04) | -86.47 | -3.27 (-3.52, -3.02) |
| Uzbekistan | 47309 (41960, 53135) | 70.37 (62.33, 79.10) | 12382 (11287, 14155) | 13.32 (12.14, 15.23) | -73.83 | -5.76 (-6.19, -5.33) |
| Vanuatu | 203 (169, 243) | 35.80 (29.74, 42.89) | 150 (107, 215) | 16.80 (11.91, 24.00) | -26.11 | -2.24 (-2.32, -2.16) |

| | | | | | | |
|---|---|---|---|---|---|---|
| Viet Nam | 95559 (88187, 104189) | 51.52 (47.52, 56.19) | 28553 (23945, 34268) | 20.05 (16.81, 24.04) | -70.12 | -2.62 (-2.97, -2.28) |
| Zambia | 63086 (59179, 67240) | 180.57 (169.34, 192.47) | 29963 (23528, 38299) | 44.72 (35.12, 57.15) | -52.5 | -4.74 (-5.00, -4.49) |
| Zimbabwe | 28601 (26072, 31269) | 79.67 (72.64, 87.15) | 21755 (13317, 34658) | 44.21 (27.00, 70.60) | -23.94 | -2.28 (-2.80, -1.76) |
| Cook Islands | 12 (11, 14) | 23.74 (21.36, 26.42) | 3 (2, 4) | 13.64 (8.59, 21.58) | -75 | -1.98 (-2.35, -1.60) |
| Niue | 2 (1, 2) | 25.58 (21.60, 30.28) | 1 (0, 1) | 24.28 (10.37, 56.57) | -50 | -0.05 (-0.53, 0.44) |
| State of Palestine | 4239 (3903, 4593) | 44.91 (41.32, 48.69) | 3856 (2658, 5642) | 26.28 (18.13, 38.41) | -9.04 | -3.08 (-3.46, -2.70) |
| High-income country | | | | | | |
| Andorra | 8 (2, 32) | 13.08 (3.22, 51.61) | 1 (0, 6) | 2.58 (0.67, 10.09) | -87.5 | -4.89 (-4.95, -4.82) |
| Antigua and Barbuda | 18 (16, 20) | 13.54 (11.96, 15.30) | 10 (7, 14) | 9.33 (6.79, 12.80) | -44.44 | -1.49 (-1.69, -1.29) |
| Australia | 2343 (2300, 2389) | 9.20 (9.02, 9.37) | 1113 (1071, 1157) | 3.67 (3.53, 3.81) | -52.5 | -2.73 (-2.90, -2.56) |
| Austria | 847 (825, 870) | 9.50 (9.26, 9.76) | 251 (233, 271) | 3.13 (2.90, 3.37) | -70.37 | -3.00 (-3.22, -2.77) |
| Bahamas | 140 (133, 147) | 23.37 (22.21, 24.60) | 55 (44, 68) | 12.69 (10.25, 15.66) | -60.71 | -1.26 (-1.65, -0.87) |
| Bahrain | 318 (307, 329) | 23.05 (22.23, 23.87) | 171 (157, 187) | 8.60 (7.89, 9.38) | -46.23 | -3.39 (-3.85, -2.92) |
| Barbados | 73 (69, 78) | 18.12 (17.00, 19.31) | 32 (20, 51) | 10.04 (6.33, 16.12) | -56.16 | -1.26 (-1.46, -1.07) |
| Belgium | 1215 (1190, 1241) | 9.99 (9.78, 10.20) | 393 (354, 432) | 3.60 (3.25, 3.96) | -67.65 | -2.87 (-3.16, -2.59) |
| British Virgin Islands | 6 (5, 7) | 22.93 (19.21, 27.29) | 4 (2, 8) | 12.73 (6.83, 23.40) | -33.33 | -1.29 (-1.46, -1.12) |
| Brunei Darussalam | 95 (90, 101) | 13.32 (12.54, 14.15) | 59 (51, 69) | 9.39 (8.07, 10.92) | -37.89 | -0.90 (-1.16, -0.65) |
| Bulgaria | 2044 (1999, 2088) | 18.35 (17.95, 18.74) | 382 (357, 408) | 6.08 (5.68, 6.50) | -81.31 | -4.00 (-4.30, -3.71) |
| Canada | 3229 (3174, 3288) | 8.25 (8.11, 8.40) | 1808 (1730, 1889) | 5.06 (4.85, 5.29) | -44.01 | -1.30 (-1.42, -1.18) |
| Chile | 5606 (5482, 5732) | 19.06 (18.64, 19.48) | 1320 (1268, 1376) | 7.16 (6.88, 7.47) | -76.45 | -2.71 (-2.99, -2.42) |
| Costa Rica | 1410 (1384, 1439) | 16.93 (16.61, 17.28) | 553 (516, 595) | 10.46 (9.75, 11.25) | -60.78 | -1.90 (-2.14, -1.66) |
| Croatia | 745 (722, 767) | 12.88 (12.48, 13.27) | 149 (137, 162) | 4.57 (4.20, 4.97) | -80 | -3.29 (-3.56, -3.02) |
| Cyprus | 166 (157, 176) | 11.13 (10.53, 11.82) | 52 (41, 66) | 3.53 (2.80, 4.54) | -68.67 | -4.37 (-4.97, -3.77) |
| Czechia | 1551 (1517, 1584) | 12.10 (11.84, 12.37) | 249 (230, 269) | 2.56 (2.37, 2.76) | -83.95 | -4.44 (-4.91, -3.97) |
| Denmark | 547 (530, 564) | 8.93 (8.65, 9.22) | 200 (179, 222) | 3.42 (3.07, 3.81) | -63.44 | -2.55 (-2.78, -2.31) |
| Estonia | 413 (400, 428) | 17.69 (17.09, 18.31) | 25 (21, 29) | 2.11 (1.79, 2.49) | -93.95 | -7.06 (-7.30, -6.82) |
| Finland | 429 (414, 445) | 6.72 (6.50, 6.98) | 102 (93, 114) | 2.29 (2.08, 2.54) | -76.22 | -0.06 (-0.42, 0.30) |
| France | 6929 (6790, 7080) | 8.98 (8.80, 9.17) | 2899 (2809, 2992) | 4.32 (4.19, 4.46) | -58.16 | -3.35 (-3.52, -3.18) |
| Germany | 7578 (7443, 7719) | 8.53 (8.38, 8.69) | 2675 (2611, 2741) | 3.65 (3.56, 3.74) | -64.7 | -6.07 (-6.50, -5.63) |
| Greece | 1142 (1114, 1172) | 10.45 (10.19, 10.72) | 282 (239, 331) | 3.74 (3.18, 4.39) | -75.31 | -3.83 (-3.99, -3.67) |
| Guyana | 1360 (1230, 1507) | 60.78 (54.91, 67.41) | 434 (244, 784) | 25.74 (14.46, 46.66) | -68.09 | -3.72 (-3.85, -3.59) |
| Hungary | 2120 (2073, 2167) | 17.18 (16.80, 17.56) | 336 (312, 362) | 3.80 (3.53, 4.10) | -84.15 | -4.56 (-4.71, -4.41) |
| Iceland | 29 (26, 32) | 6.29 (5.73, 6.94) | 11 (9, 15) | 2.57 (1.94, 3.40) | -62.07 | -2.82 (-3.21, -2.42) |

| | | | | | | |
|---|---|---|---|---|---|---|
| Ireland | 492 (475, 511) | 9.18 (8.85, 9.53) | 205 (181, 233) | 3.80 (3.37, 4.33) | -58.33 | -3.16 (-3.46, -2.86) |
| Israel | 1061 (1038, 1085) | 11.54 (11.30, 11.81) | 588 (562, 618) | 3.39 (3.23, 3.55) | -44.58 | -3.72 (-3.92, -3.52) |
| Italy | 5457 (5353, 5565) | 9.66 (9.47, 9.85) | 1089 (1049, 1132) | 2.76 (2.65, 2.87) | -80.04 | -3.63 (-3.87, -3.39) |
| Japan | 7707 (7566, 7849) | 6.32 (6.21, 6.44) | 1858 (1799, 1918) | 2.36 (2.29, 2.44) | -75.89 | -3.23 (-3.34, -3.11) |
| Republic of Korea | 10504 (9935, 11087) | 15.67 (14.82, 16.54) | 694 (650, 742) | 2.76 (2.58, 2.95) | -93.39 | -5.10 (-5.41, -4.80) |
| Kuwait | 937 (909, 964) | 17.29 (16.79, 17.79) | 428 (404, 456) | 8.75 (8.25, 9.31) | -54.32 | -2.06 (-2.17, -1.94) |
| Latvia | 656 (635, 679) | 17.02 (16.47, 17.61) | 46 (38, 55) | 2.96 (2.48, 3.52) | -92.99 | -5.97 (-6.32, -5.62) |
| Lithuania | 862 (835, 889) | 15.21 (14.74, 15.68) | 75 (64, 87) | 3.38 (2.88, 3.95) | -91.3 | -5.18 (-5.40, -4.96) |
| Luxembourg | 41 (38, 45) | 8.75 (8.09, 9.49) | 16 (12, 20) | 2.34 (1.82, 2.99) | -60.98 | -4.00 (-4.41, -3.60) |
| Malta | 65 (60, 69) | 11.35 (10.60, 12.17) | 23 (19, 29) | 5.50 (4.46, 6.76) | -64.62 | -1.81 (-2.04, -1.58) |
| Monaco | 3 (3, 3) | 7.75 (6.99, 8.60) | 1 (1, 2) | 2.74 (1.66, 4.52) | -66.67 | -2.89 (-2.99, -2.78) |
| Nauru | 20 (16, 25) | 67.40 (53.23, 85.58) | 3 (2, 5) | 8.94 (5.14, 15.86) | -85 | -5.75 (-6.09, -5.40) |
| Netherlands (Kingdom of the) | 1618 (1590, 1647) | 8.32 (8.17, 8.46) | 675 (646, 704) | 3.96 (3.79, 4.13) | -58.28 | -2.38 (-2.56, -2.19) |
| New Zealand | 650 (635, 667) | 11.16 (10.88, 11.45) | 276 (242, 316) | 4.68 (4.10, 5.37) | -57.54 | -2.29 (-2.45, -2.14) |
| Norway | 511 (495, 529) | 8.67 (8.39, 8.97) | 122 (111, 135) | 2.36 (2.14, 2.60) | -76.13 | -3.77 (-3.97, -3.57) |
| Oman | 2507 (2182, 2885) | 39.10 (34.01, 45.03) | 851 (747, 967) | 10.40 (9.14, 11.82) | -66.06 | -3.52 (-4.10, -2.94) |
| Palau | 12 (10, 14) | 35.32 (30.64, 41.13) | 4 (2, 7) | 22.30 (12.58, 37.33) | -66.67 | -1.44 (-1.85, -1.04) |
| Panama | 1984 (1737, 2231) | 30.45 (26.65, 34.28) | 952 (896, 1012) | 13.28 (12.50, 14.12) | -52.02 | -2.45 (-2.55, -2.34) |
| Poland | 9756 (9555, 9951) | 17.39 (17.03, 17.73) | 1393 (1344, 1445) | 4.35 (4.20, 4.51) | -85.72 | -4.51 (-4.82, -4.20) |
| Portugal | 1739 (1705, 1776) | 14.71 (14.42, 15.02) | 269 (250, 289) | 3.16 (2.94, 3.40) | -84.53 | -4.53 (-5.04, -4.01) |
| Qatar | 213 (199, 227) | 20.81 (19.48, 22.18) | 175 (161, 190) | 6.02 (5.55, 6.53) | -17.84 | -3.46 (-3.59, -3.32) |
| Romania | 10826 (10568, 11089) | 31.39 (30.64, 32.15) | 1230 (1173, 1287) | 6.63 (6.33, 6.95) | -88.64 | -5.18 (-5.43, -4.93) |
| Russian Federation | 47084 (46218, 47958) | 21.66 (21.26, 22.06) | 6036 (5370, 6775) | 4.50 (4.00, 5.05) | -87.18 | -5.04 (-5.41, -4.68) |
| San Marino | 3 (2, 5) | 12.71 (7.39, 21.23) | 0 (0, 1) | 1.43 (0.67, 3.08) | -100 | -6.44 (-6.71, -6.17) |
| Saudi Arabia | 17600 (14727, 20946) | 44.39 (37.09, 52.91) | 3201 (2253, 4626) | 6.16 (4.33, 8.90) | -81.81 | -5.83 (-5.93, -5.73) |
| Seychelles | 27 (25, 30) | 16.30 (14.86, 17.89) | 25 (19, 32) | 14.25 (10.97, 18.50) | -7.41 | -0.04 (-0.19, 0.11) |
| Singapore | 370 (355, 385) | 7.66 (7.36, 7.99) | 96 (80, 115) | 2.07 (1.72, 2.47) | -74.05 | -3.26 (-3.65, -2.86) |
| Slovakia | 1177 (1151, 1203) | 14.73 (14.41, 15.06) | 323 (303, 344) | 6.08 (5.70, 6.48) | -72.56 | -2.88 (-3.12, -2.65) |
| Slovenia | 241 (231, 250) | 10.38 (9.98, 10.77) | 41 (35, 48) | 2.25 (1.95, 2.62) | -82.99 | -4.66 (-4.92, -4.39) |
| Spain | 3790 (3718, 3862) | 9.16 (8.99, 9.34) | 1059 (999, 1124) | 3.12 (2.94, 3.31) | -72.06 | -3.29 (-3.54, -3.04) |
| Saint Kitts and Nevis | 29 (27, 32) | 30.31 (27.59, 33.23) | 9 (6, 14) | 16.28 (10.35, 25.47) | -68.97 | -1.74 (-1.96, -1.52) |
| Sweden | 814 (792, 839) | 6.96 (6.76, 7.16) | 256 (238, 275) | 2.47 (2.30, 2.66) | -68.55 | -2.79 (-3.05, -2.54) |
| Switzerland | 658 (640, 679) | 8.17 (7.94, 8.43) | 328 (308, 349) | 3.94 (3.71, 4.20) | -50.15 | -2.05 (-2.22, -1.88) |

| | | | | | | |
|---|---|---|---|---|---|---|
| Trinidad and Tobago | 889 (763, 1013) | 32.51 (27.89, 37.05) | 311 (201, 492) | 19.07 (12.30, 30.22) | -65.02 | -1.56 (-1.62, -1.51) |
| Turks and Caicos Islands | 6 (4, 9) | 18.61 (12.64, 27.60) | 3 (1, 8) | 5.61 (1.78, 16.48) | -50 | -3.36 (-3.62, -3.11) |
| United Arab Emirates | 682 (631, 738) | 15.14 (14.01, 16.38) | 496 (436, 564) | 5.03 (4.42, 5.72) | -27.27 | -2.79 (-2.97, -2.61) |
| United Kingdom of Great Britain and Northern Ireland | 7298 (7158, 7448) | 9.30 (9.12, 9.49) | 3073 (2842, 3331) | 4.46 (4.12, 4.83) | -57.89 | -2.21 (-2.38, -2.04) |
| United States of America | 45985 (45202, 46840) | 11.23 (11.04, 11.44) | 23989 (23295, 24711) | 6.48 (6.29, 6.67) | -47.83 | -1.61 (-1.73, -1.49) |
| Uruguay | 1311 (1284, 1336) | 23.66 (23.18, 24.12) | 226 (206, 249) | 6.66 (6.07, 7.34) | -82.76 | -4.10 (-4.21, -4.00) |
| Anguilla | 3 (2, 5) | 19.04 (13.04, 27.66) | 1 (0, 2) | 5.77 (2.83, 11.74) | -66.67 | -3.40 (-3.52, -3.28) |
| Montserrat | 4 (3, 6) | 20.38 (15.00, 27.68) | 0 (0, 1) | 6.47 (2.50, 17.37) | -100 | -3.38 (-3.42, -3.35) |

CI: confidence interval; EAPC: estimated annual percentage change; U5MR: under-5 mortality rate; UI: uncertainty interval.
