# Supplementary material for: Tracking under-five mortality from 1990 to 2023: Global, regional, and national trends, inequities, and projections toward achieving SDG Target 3.2 by 2030
Source: PLoS One. 2026 Apr 1;21(4):e0343745. doi: 10.1371/journal.pone.0343745 (PMC13042728; doi:10.1371/journal.pone.0343745)
Supplement: S3 Table — U5MR: under-5 mortality rate. (PDF) [file pone.0343745.s003.pdf]

S3 Table. Years in which U5MR reduction target is expected to be achieved at the national level

Region	Year
Afghanistan	2045
Albania	2023
Algeria	2023
Andorra	2023
Angola	2045
Anguilla	2023
Antigua and Barbuda	2023
Argentina	2023
Armenia	2023
Australia	2023
Austria	2023
Azerbaijan	2023
Bahamas	2023
Bahrain	2023
Bangladesh	2027
Barbados	2023
Belarus	2023
Belgium	2023
Belize	2023
Benin	2073
Bhutan	2023
Bolivia (Plurinational State of)	2023
Bosnia and Herzegovina	2023
Botswana	2053
Brazil	2023
British Virgin Islands	2023
Brunei Darussalam	2023
Bulgaria	2023
Burkina Faso	2059
Burundi	2039
Cabo Verde	2023
Cambodia	2023
Cameroon	2053
Canada	2023
Central African Republic	2357
Chad	2086
Chile	2023
China	2023
Colombia	2023
Comoros	2037
Congo	2038
Cook Islands	2023

Costa Rica	2023
Croatia	2023
Cuba	2023
Cyprus	2023
Czechia	2023
Côte d'Ivoire	2059
Democratic People's Republic of Korea	2023
Democratic Republic of the Congo	2059
Denmark	2023
Djibouti	2051
Dominica	Deteriorating
Dominican Republic	2038
Ecuador	2023
Egypt	2023
El Salvador	2023
Equatorial Guinea	2058
Eritrea	2031
Estonia	2023
Eswatini	2054
Ethiopia	2037
Fiji	2279
Finland	2023
France	2023
Gabon	2033
Gambia	2037
Georgia	2023
Germany	2023
Ghana	2034
Greece	2023
Grenada	2023
Guatemala	2023
Guinea	2073
Guinea-Bissau	2050
Guyana	2025
Haiti	2052
Honduras	2023
Hungary	2023
Iceland	2023
India	2027
Indonesia	2023
Iran (Islamic Republic of)	2023
Iraq	2023
Ireland	2023
Israel	2023

Italy	2023
Jamaica	2023
Japan	2023
Jordan	2023
Kazakhstan	2023
Kenya	2036
Kiribati	2087
Kosovo (UNSCR 1244)	2023
Kuwait	2023
Kyrgyzstan	2023
Lao People's Democratic Republic	2034
Latvia	2023
Lebanon	2023
Lesotho	2088
Liberia	2047
Libya	2029
Lithuania	2023
Luxembourg	2023
Madagascar	2056
Malawi	2030
Malaysia	2023
Maldives	2023
Mali	2067
Malta	2023
Marshall Islands	2033
Mauritania	2034
Mauritius	2023
Mexico	2023
Micronesia (Federated States of)	2023
Monaco	2023
Mongolia	2023
Montenegro	2023
Montserrat	2023
Morocco	2023
Mozambique	2043
Myanmar	2036
Namibia	2049
Nauru	2023
Nepal	2025
Netherlands (Kingdom of the)	2023
New Zealand	2023
Nicaragua	2023
Niger	2066
Nigeria	2087

Niue	2023
North Macedonia	2023
Norway	2023
Oman	2023
Pakistan	2057
Palau	2023
Panama	2023
Papua New Guinea	2045
Paraguay	2023
Peru	2023
Philippines	2027
Poland	2023
Portugal	2023
Qatar	2023
Republic of Korea	2023
Republic of Moldova	2023
Romania	2023
Russian Federation	2023
Rwanda	2031
Saint Kitts and Nevis	2023
Saint Lucia	2023
Saint Vincent and the Grenadines	2023
Samoa	2023
San Marino	2023
Sao Tome and Principe	2023
Saudi Arabia	2023
Senegal	2033
Serbia	2023
Seychelles	2023
Sierra Leone	2064
Singapore	2023
Slovakia	2023
Slovenia	2023
Solomon Islands	2023
Somalia	2095
South Africa	2038
South Sudan	2067
Spain	2023
Sri Lanka	2023
State of Palestine	2025
Sudan	2046
Suriname	2023
Sweden	2023
Switzerland	2023

Syrian Arab Republic	2023
Tajikistan	2025
Thailand	2023
Turkey	2023
Timor-Leste	2037
Togo	2052
Tonga	2023
Trinidad and Tobago	2023
Tunisia	2023
Turkmenistan	2042
Turks and Caicos Islands	2023
Tuvalu	2023
Uganda	2032
Ukraine	2023
United Arab Emirates	2023
United Kingdom of Great Britain and Northern Ireland	2023
United Republic of Tanzania	2032
United States of America	2023
Uruguay	2023
Uzbekistan	2023
Vanuatu	2023
Venezuela (Bolivarian Republic of)	2023
Viet Nam	2023
Yemen	2035
Zambia	2035
Zimbabwe	2048

U5MR: under-5 mortality rate.
